# Guided Zygomatic Implantology for Oral Cancer Rehabilitation: A Case Report

**DOI:** 10.3390/jcm12113653

**Published:** 2023-05-24

**Authors:** Francesco Grecchi, Roberto Giuseppe D’Ambrogio, Luigi Vito Stefanelli, Fabrizio Grivetto, Funda Goker, Massimo Del Fabbro, Alberto Schreiber, Cesare Piazza, Stefano Salgarello, Camilla Dosio, Emma Grecchi

**Affiliations:** 1 Private Practice, Via Boccaccio 34, 20123 Milan, Italy; dr.grecchi@tiscali.it (F.G.);; 2 Department of Surgical Specialties, Dental Clinic, School of Dentistry, University of Brescia, Piazza Spedali Civili 1, 25123 Brescia, Italy; r.dambrogio@libero.it (R.G.D.); stefano.salgarello@unibs.it (S.S.); 3 Private Practice, Viale Leonardo Da Vinci 256, 00145 Rome, Italy; gigistef@libero.it; 4 Azienda Ospedaliero Universitaria Maggiore Della Carità Largo Bellini, 28100 Novara, Italy; info@curadentis.it (F.G.); camilladosio@gmail.com (C.D.); 5 Department of Biomedical, Surgical and Dental Sciences, University of Milano, 20122 Milan, Italy; 6 Dental and Maxillo-Facial Surgery Unit, Fondazione IRCCS Ca’ Granda Ospedale Maggiore Policlinico, 20122 Milan, Italy; 7 Department of Surgical Specialties, Ear, Nose and Throat Clinic, School of Dentistry, University of Brescia, Piazza Spedali Civili 1, 25123 Brescia, Italy; albertoschreiber82@gmail.com (A.S.); ceceplaza@libero.it (C.P.)

**Keywords:** virtual surgical planning, oncologic patients, oral rehabilitation, zygomatic implants, immediate loading, adenoid cystic carcinoma, anterolateral thigh free flap, computer-aided implantology

## Abstract

Oral rehabilitation after maxillary oncological resection is challenging. This case report presents the rehabilitation of a 65-year-old Caucasian male adenoid cystic carcinoma patient using a myo-cutaneous thigh flap, zygomatic implant placement, and an immediate fixed provisional prosthesis made with computer-aided technologies. The patient presented complaints of asymptomatic enlarged swelling of 5-mm on the right hard hemi-palate. There was an oro-antral communication deriving from a previous local excision. Preoperative radiographs showed the involvement of the right maxilla, maxillary sinus, and nose with a suspect involvement of the maxillary division of the trigeminal nerve. Treatment was planned through a fully digital workflow. A partial maxillectomy was performed endoscopically, and maxilla was reconstructed using an anterolateral thigh free flap. Two zygomatic implants were inserted simultaneously. A provisional fix full-arch prosthesis was manufactured preoperatively through a fully digital workflow and was placed in the operating room. Following post-operative radiotherapy, the patient received a final hybrid prosthesis. During the follow-up period of two years, the patient reported good function, aesthetics, and significant enhancement in quality of life. According to the results of this case, the protocol represented can be a promising alternative for oral cancer patients with large defects, and can lead to an improved quality of life.

## 1. Introduction

Dental rehabilitation after maxillary oncological resection is a demanding clinical condition with therapeutic limits and technical difficulties deriving from the surgical alteration of the anatomy of the region. The big challenge is to re-establish the physiologic anatomical separation between the oral cavity and the nasal/paranasal region, and rehabilitate the oral function with a prosthesis that can restore satisfactory facial aesthetics [1,2,3,4,5].

In the past, rehabilitation was mainly obtained with a removable obturator prosthesis, which was effective in several clinical situations. However, this solution was not sufficient when the resection involved a large amount of soft palate, more than one third of the hard palate, and maxilla where there were not sufficient teeth to retain the prosthesis efficiently [4]. As alternatives, pedicled or free muscle flaps have been proposed, and were successfully applied to separate the oral cavity from the nasal/paranasal region. However, these were not adequate to provide both functional and aesthetical results [5]. The reconstructive procedures with osteo-myo-cutaneous composite flaps aimed to solve these limits in an attempt to recreate a sufficient amount of bone volume to place endosseous implants as anchorage for the dental prosthesis [6,7,8,9,10]. However, these techniques had several limits in the maxilla, since the shape of the defect often impedes a satisfactory bone volume reconstruction for implant placement. Additionally, they represent other disadvantages, such as long duration of therapy, several interventions from oncologic resection to patient rehabilitation, and additional risks, especially in cases of post-operative radiotherapy [5,6,7,9,10,11,12].

Zygomatic implants (ZI) were proposed by Brånemark in 1988 for the treatment of oncologic patients, initially as anchorage for obturator prostheses. In the following years, ZIs had been used with different protocols with/without pedicled or myo-cutaneous revascularized free flaps with promising results [13,14,15,16,17,18,19,20,21,22,23,24,25,26,27]. A recent review of the literature reported successful outcomes despite the possible negative effects of radiotherapy [19]. Another important point that has emerged is the timing of implant loading, since it is possible to perform immediate loading procedures in oncologic patients by the utilization of zygomatic implants with a net and concrete reduction of treatment time necessary from cancer removal to a complete rehabilitation of the patient [20,23,24].

The evolution of computer-aided design/computer-aided manufacturing (CAD/CAM) technologies has demonstrated that three-dimensional virtual surgical planning is a very useful tool for the maxillofacial surgical reconstructions, and in the rehabilitation of edentulous patients via digital immediate loading procedures [10,28,29,30,31,32,33,34,35]. Scientific publications show that CAD/CAM technologies can effectively reduce the treatment time, invasiveness, and costs, with increased precision. However, there are a limited number of reports about well-described full-digital therapeutic protocols in the oral oncologic field [10,30,31,32,33,34,35].

The aim of this case report was to present a fully digital one-day rehabilitation protocol for the treatment of an adenoid cystic carcinoma patient using a revascularized myo-cutaneous thigh flap, guided zygomatic implants insertion, and an immediately loaded fixed provisional prosthesis made with computer-aided technologies.

## 2. CASE Presentation

The treatment was performed in accordance with the principles embedded in The Declaration of Helsinki of the World Medical Association for human biomedical research. Being a single case report, no ethical clearance was needed.

## 3. Patient History

A 65-year-old Caucasian male patient presented complaints of clearly visible, asymptomatic enlarged swelling of about 5-mms on the right hard hemi-palate; the lesion was asymptomatic and there were no signs of facial paralysis. On the first visit, an incisional biopsy was performed under local anesthesia to identify the nature of the lesion. The results of the histological examination showed a neoplasm with a morphology that indicated an immunophenotype of salivary origin, which supported the first suspicion for a benign/low grade of malignancy.

The lesion was removed as 6-mms to obtain healthy margins. The resection created an oro-antral communication of about 1.5 cm. The final histopathological diagnosis indicated an adenoid cystic carcinoma with positive margins.

### 3.1. Detailed Medical History

A CT (Computed Tomography) scan was requested at this point, and the results showed bone lysis localized on the palatine and maxillary bone neighboring the lesion. An MR (Magnetic resonance) exam with contrast for the head and neck region showed a residual focal alteration of the hard palate on the right side. The lesion involved posteriorly a part of the soft palate, extending until the borders of the medial part and to the floor of the homolateral maxillary sinus laterally, marking until the oral cavity inferiorly; in the cranial aspect, it was causing a reduction of the aero lumen and blocking the floor of the nasal concha.

**Figure 1:**
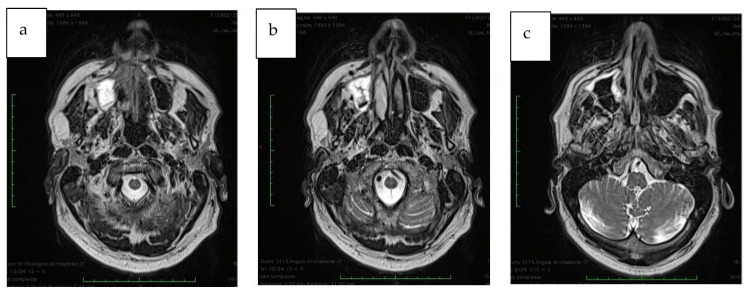
(**a**–**c**) MRI section showing the extension of the maxillary lesion.

The approximate dimensions of the neoplasm: 3.3 cm anteroposterior, 2 cm transverse, and 2.6 cm cranio-caudal. The margins of the lesion were well-defined; however, the involvement of the CNV2 nerve ascendent tract (maxillary division of the trigeminal nerve) (Figure 1a–c) was suspected.

**Figure 2:**
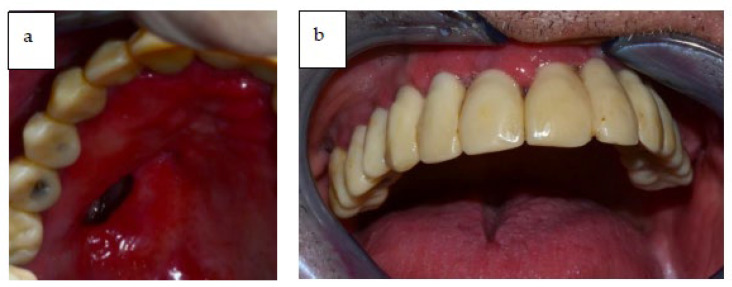
(**a**,**b**) Clinical intra-oral view of the patient showing extensive oro-antral communication.

Anamnesis of the patient: Body mass index: 31.35. Past medical history: ASA 2. Previous surgeries: Inguinal hernioplasty and tonsillectomy operation. No pharmacological history and no allergies. Former tobacco smoker. An extensive oro-antral communication was present from the previous operation, with a residual palatal-maxillary defect (Figure 2a,b). The defect was Class 3 according to Urken’s Modified Okay Classification System [35]. As a result, the confirmed diagnosis was right maxillary adenoid cystic carcinoma with perineural and CNV2 invasion in the pterygo-palatine fossa.

At this point, the patient was referred to the clinic of the authors of this study (Spedali Civili di Brescia—ENT Clinic, Dental Clinic). The patient had a comprehensive evaluation, and surgery was planned as: right subtotal maxillectomy, reconstruction with a free flap of the right thigh, and guided placement of zygomatic implants with immediate loading.

### 3.2. Preoperative Oral Situation

The patient reported to have lost teeth #32, #33, and #34 due to root fracture and periodontal disease two months before the oncological resection, as can be seen in Figure 2a,b (that were placed through a removable prosthesis).

**Figure 3:**
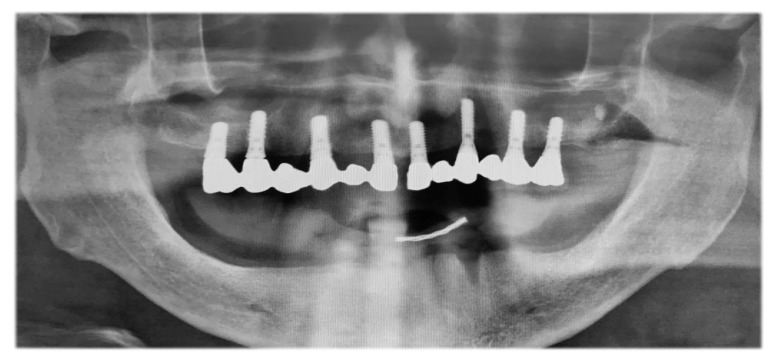
Preoperative panoramic radiograph.

The patient presented a fixed maxillary prosthesis on 8 ITI dental implants that were inserted 15 years ago, and had total edentulous in the mandible (Figure 3).

### 3.3. Preoperative Protocol

The preoperative regimen included: a test for hereditary hemochromatosis, a pulmonary X-ray, and an ECG. The results from these tests did not show any obstacle for surgery, and the intervention was planned as resection of the neoformation with local palatoplasty.

### 3.4. Preoperative Digital Planning

Digital impressions of the upper and lower jaws were taken and matched with the CT scan of the patient, with the aim to create a digital model and to develop a digital project of the rehabilitation.

**Figure 4:**
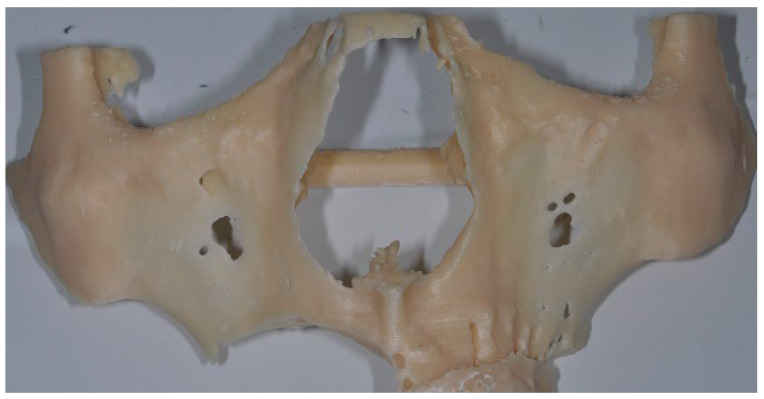
Surgical osteotomies planned for tumor excision were transferred to the 3D printed digital model.

The surgical osteotomies that were planned for removal of the tumor were transferred to the digital project (Figure 4) through a dedicated software for the digital planning of implant surgery, and were printed (EZgoma^©^, Noris Medical Ltd., Nesher, Israel).

**Figure 5:**
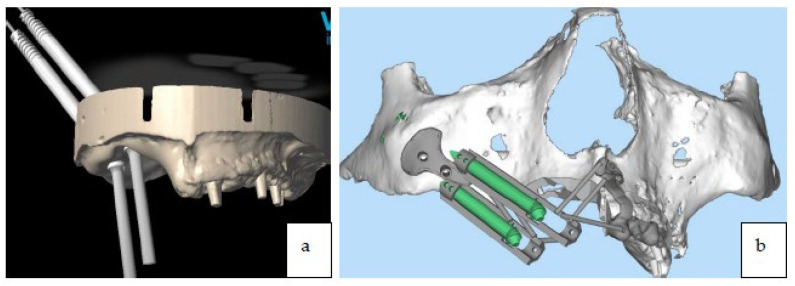
(**a**,**b**) Preoperative digital planning of implant placement.

**Figure 6:**
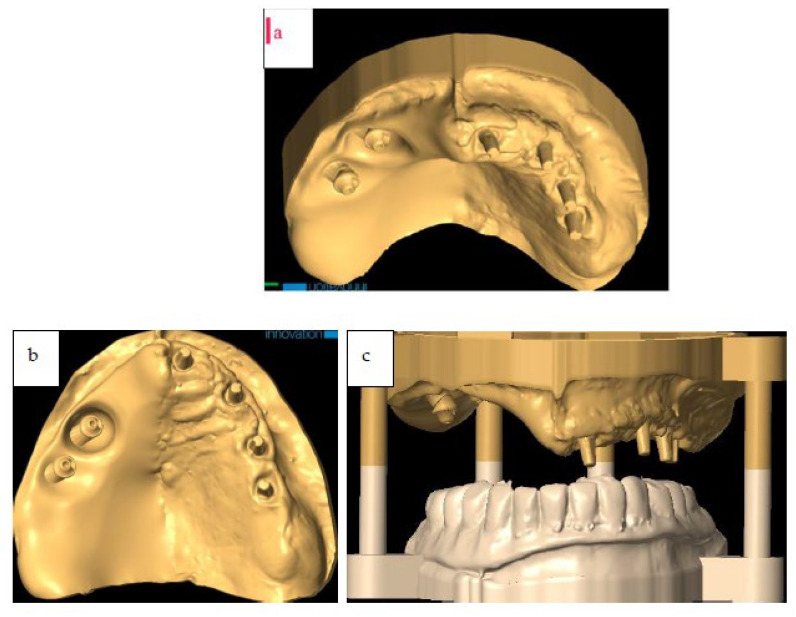
(**a**,**b**) Digital model with implants. (**c**) Digital model with implants mounted with inferior jaw.

**Figure 7:**
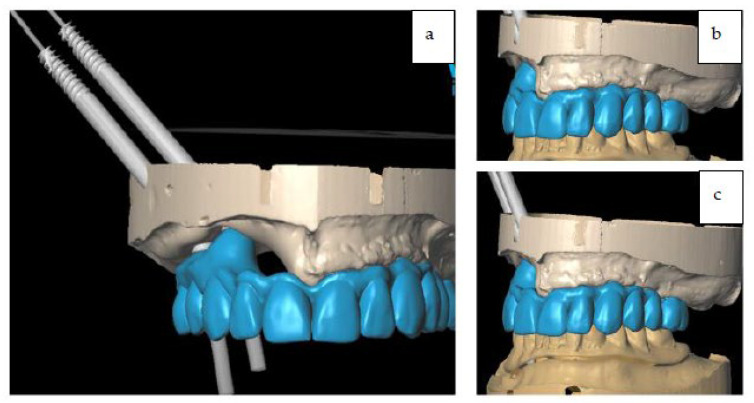
(**a**) Digital project of immediate loading full-arch provisional. (**b,c**) Digital project of immediate loading full-arch provisional in occlusion.

The surgery was programmed as: two zygomatic implant placements (length 45 mm, diameter 4.2 mm, Noris implant^©^) fixed on the right zygoma with emergence on sites #13 and #15), and a fix provisional full-arch prosthesis (Figure 5a,b, Figure 6a–c and Figure 7a–c).

**Figure 8:**
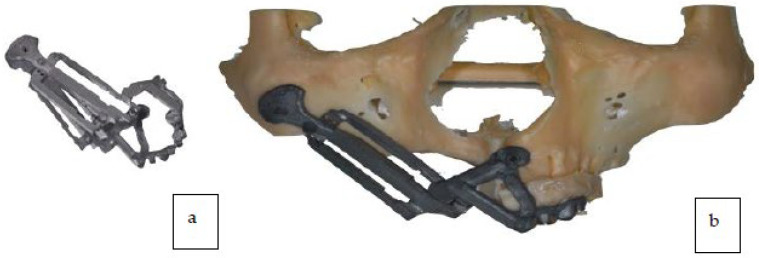
(**a**) Titanium guide for zygomatic implant placement. (**b**) Titanium guide for zygomatic implant placement on stereolithographic model.

A titanium surgical guide was manufactured using CAD/CAM technologies for the placement of the zygomatic implants along with a reinforced provisional prosthesis (titanium and Poly-methyl methacrylate (PMMA) material produced by milling with a 5-axis machine) (Figure 8a,b).

**Figure 9:**
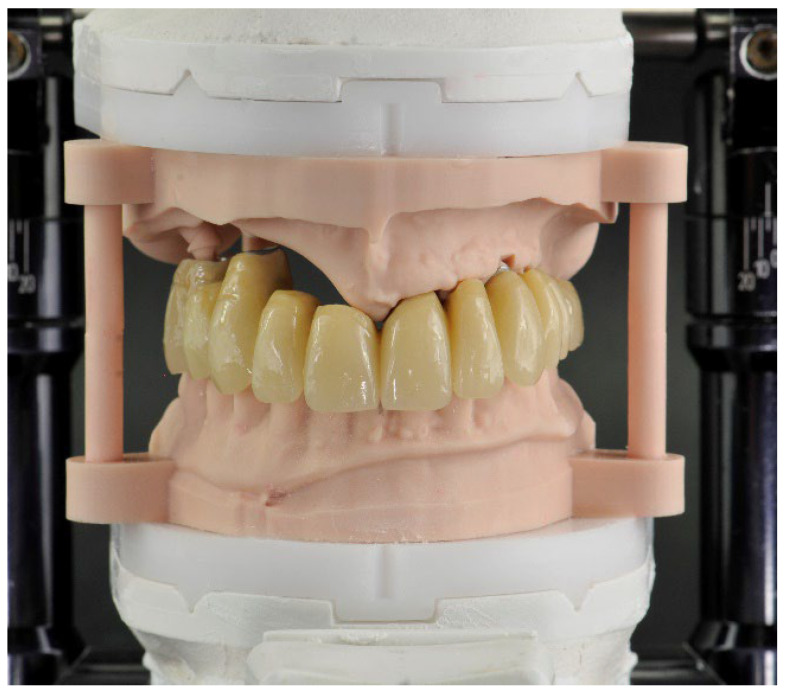
Immediate provisional prosthesis in Titanium and PMMA articulated with the inferior arch.

The details of this procedure were reported in previous publications by the same team of authors of this work [25,26]. The 3D printed model used in this work allowed checking of the final occlusion, and finishing/polishing the provisional prosthesis (Figure 9).

### 3.5. Pre-Surgical Medication

Antibiotic prophylaxis: Unasyn (intra-venous—2 g Ampisilin/1 g sulbactam—q 6 h 7 days).

PONV (post-operative nausea and vomiting): Ondansetron 8 mg + Droperidol 1.25 mg.

Analgesic: Paracetamol 1 g, Ketorolac 30 mg, Morphine 10 mg.

## 4. Surgical Technique

**Figure 10:**
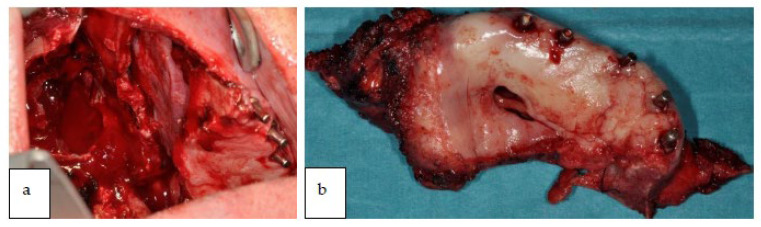
(**a**) Intra-oral view of the resected site. (**b**) Tumor resected.

The patient was operated under general anesthesia (with Propofol and Ramifentanil). The duration of intervention was 10 h. The surgery started with tracheostomy and endoscopic right subtotal maxillectomy (Figure 10a,b).

**Figure 11:**
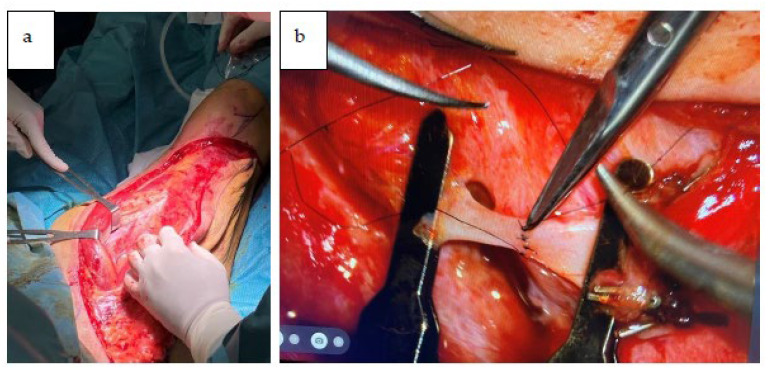
(**a**) Harvesting of ALT flap. (**b**) Microsurgical anastomosis between facial artery and artery of ALT flap.

A new maxilla was reconstructed with the ALT right free flap that was revascularized using facial vessels (Figure 11a,b).

Following the tumor excision, two surgical teams started working simultaneously; one performing the harvesting of the right anterolateral thigh free flap (ALT), and the other working intra-orally for reconstruction. The resection included the left premaxilla because of the presence of neoplastic tissue extending in the left nasal cavity. Due to the remote location of the zygomatic implants, the placement and fixing of the surgical guide were possible without any problems, despite the large dimensions of the defect.

**Figure 12:**
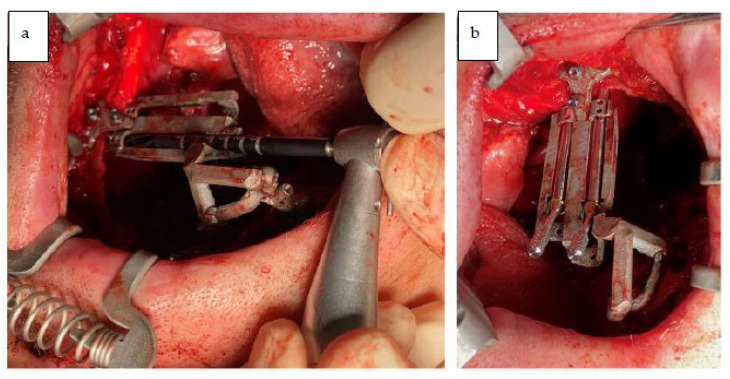
(**a**) Zygomatic implant guided osteotomy. (**b**) Zygomatic implants placement.

Two zygomatic implants were placed in the right zygomatic bone (45 mm and 52.5 mm Noris medical) with insertion torque > 80 N cm (Figure 12a shows placement of the surgical guide and drilling; Figure 12b shows 2 zygomatic implants inserted).

**Figure 13:**
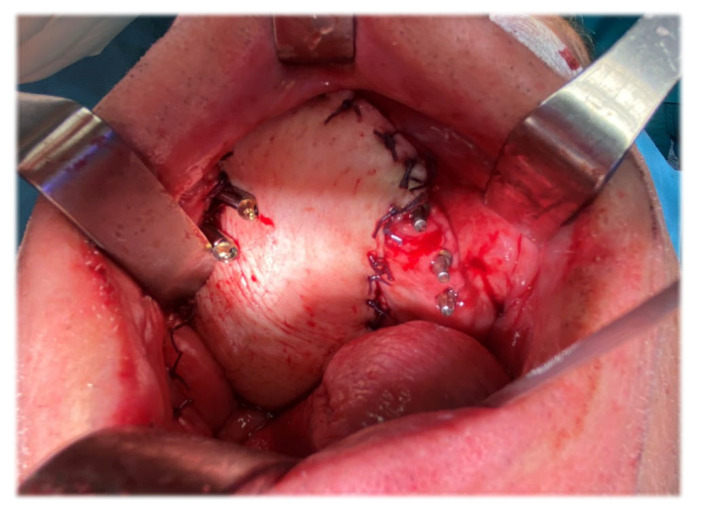
Intra-oral view of the patient showing reconstruction with vascularized right ALT flap. [Note: the reconstruction of a much larger area than the preoperatively planned resection (due to presence of neoplastic tissue in the left nasal cavity) with the remaining dental implants on left maxillary side #23, #25, #26].

Figure 13 shows an intra-oral view of the patient showing reconstruction with the vascularized right ALT flap. The antero-lateral thigh revascularized soft tissue flap was placed medially to the zygomatic implants and was sutured to the margins of the resection, as can be seen in Figure 13. As a result, the smooth body of the implants were surrounded by cheek mucosa on the lateral aspect, and the soft tissue flap on the medial aspect.

**Figure 14:**
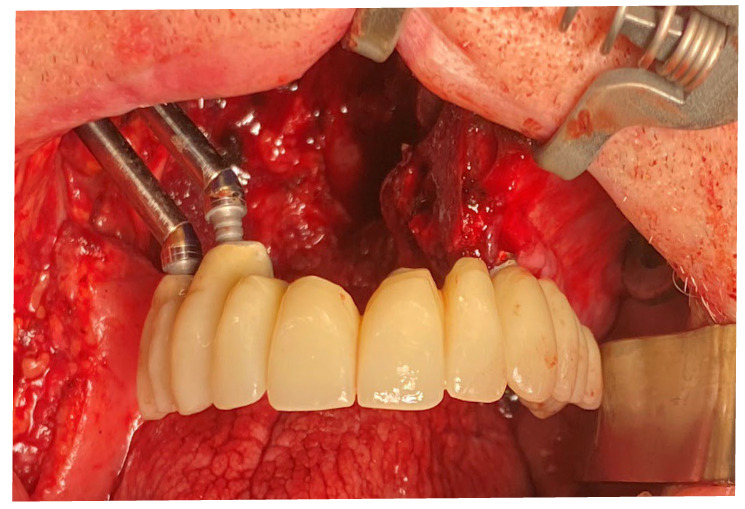
Full-arch upper provisional in place.

Multi-unit abutments (MUA) and provisional titanium abutments were placed accordingly with the digital project. The provisional prosthesis also served for checking the precision of the intervention performed. After verification, the provisional prosthesis was connected to the titanium provisional abutment that was screwed onto the zygomatic implants (Figure 14).

**Figure 15:**
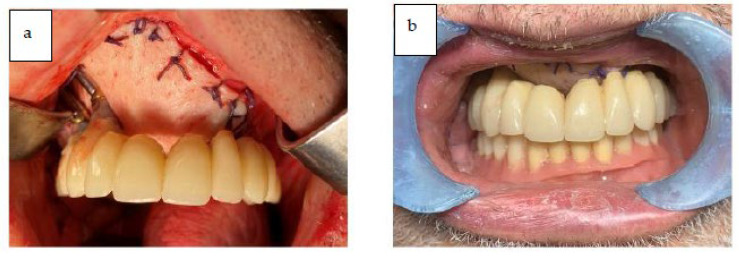
(**a**,**b**) The provisional prosthesis in occlusion.

After the reconstructive phase with the ALT flap was completed, the patient had the provisional prosthesis placed, and left the operating room with rehabilitation completed in one day. The fixed provisional prosthesis was delivered to the patient on the day of the surgery (Figure 15).

### Post-Operative Report

**Figure 16:**
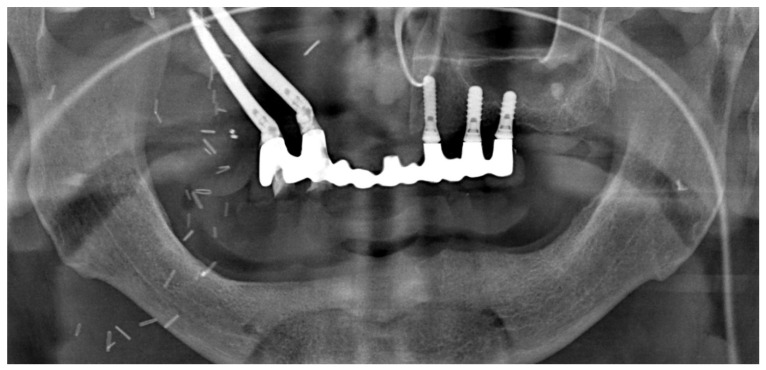
Panoramic X-ray at 7 days after surgery.

No post-operative complications were seen, and the patient was discharged from the hospital after 10 days. The patient was able to return to a normal diet (solid) after just 7 days following surgery, with no further complaints regarding function or pain, apart from the residual swelling caused by the intervention (Figure 16 shows the orthopantomogram view of the rehabilitation 7 days after surgery). Figure 16 shows the provisional prosthesis in place at 7 days follow-up (Note on Figure 16: In the radiological view, the provisional prosthesis shows some misfits with the implants on the right side of the maxilla (second quadrant). This is because it was relined and was adapted with a thin layer of acrylic resin to provide a stable and precise fit bilaterally on the implants).

**Figure 17:**
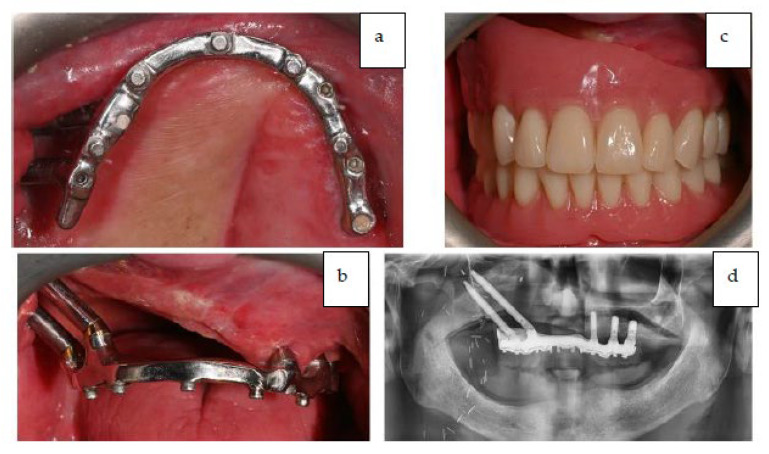
(**a**–**c**) Intra-oral occlusal and frontal view of the titanium bar retaining the definitive prosthesis and final prosthesis in occlusion. (**d**) Panoramic X-ray showing final prosthetic rehabilitation in place.

The occlusion and prosthesis were checked at a follow up on the 7th post-operative day. The patient underwent a cycle of radiotherapy without any complications or dehiscence of the flaps. The final fixed prosthesis was delivered 8 months after the surgical intervention. The definitive final removable prosthesis was retained by an implant-supported titanium bar that allowed the patient to have a strong and stable occlusion during function and provided easy access for oral hygiene. The patient reported a complete satisfaction from phonetic, functional, and aesthetic points of view. Figure 17a–d shows the final prosthesis (Note on Figure 17: The evolution of the oro-naso-sinusal defect repair through the soft tissue graft was uneventful without any complications. As can be seen in Figure 17, the tissue was œdematic for the first weeks and slowly shrank to healthy dimensions after healing).

On the follow-up appointment at 12 months after surgery, the patient presented no signs of recurrence of the disease. There were no complications or discomfort.

**Figure 18:**
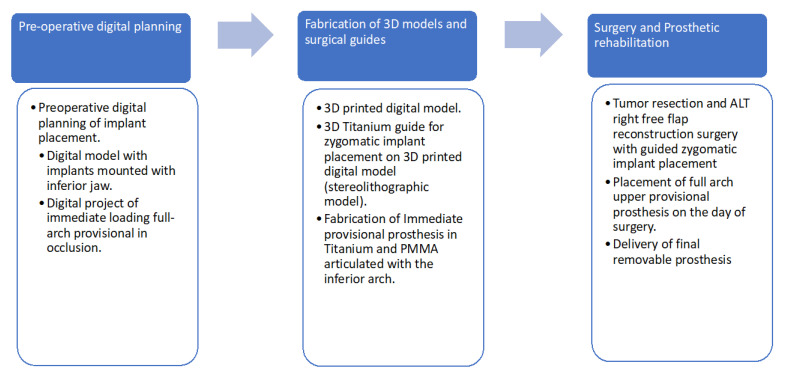
Oral rehabilitation steps of the patient. PMMA: Poly-methyl methacrylate; ALT: antero-lateral thigh.

A brief explanation of the treatment steps can be seen in Figure 18.

The patient deceased after 2 years following surgery. During the follow-up period of these two years (with scheduled appointments every 6 months), the patient reported good function, aesthetics, and a significant enhancement in quality of life.

## 5. Discussion

The primary goal of oral cancer treatment is not just the reconstruction of the palatomaxillary defect, but a total oral rehabilitation with harmony and function [5,10,36,37]. A surgical reconstructive option that can prevent the restoration of the dentition should be avoided whenever possible [5,10]. It should be taken under consideration that, these patients often have a critical prognosis with shorter life expectations, and the providing of a quality of life (QoL) in short time becomes more important. In such situations, zygomatic implants can make a crucial improvement in QoL by shortening treatment time and improving the level of positive results. This underlies the fact that the zygomatic bone very often remains healthy without any metastasis, due to its anatomical features, and represents an alternative anchorage for implants even after major resections in the maxillary bone [21,22,35].

The final rehabilitation of the maxilla can be obtained with different techniques, and all the techniques represent some advantages, disadvantages, and success rates [27,36,37,38,39,40,41,42,43,44,45,46,47,48,49,50,51,52,53,54,55,56,57,58,59,60]. There are studies that report the superiority of the flaps with respect to the obturator plate for their stability and fundamental function of separation between the nasal sinus and buccal compartments [49,52]. There are also studies that describe a better QoL in patients rehabilitated with an obturator prosthesis, since this option represents a faster maxillary rehabilitation than flaps [49,50,51,52]. The planning for the type of therapy mostly depends on the classification of the maxillectomy to be performed, and Okay et al. in 2001 proposed a classification that focusses on the potential residual function of the unresected maxilla from a rehabilitation point of view [4]. In cases of small defects, the reconstruction choice usually focusses on the use of local flaps. In larger defects, free flaps and/or various types of prosthetics (including obturators) are utilized mostly for reconstruction [52,53,54]. However, for very large defects (Okay’s Class 3), free flaps are not sufficient to achieve an adequate level of quality of life for the patient, and additional techniques are needed [22,23,24,25,26,27,28,29,30,31,32,33,34,35,36,37].

The recent developments in reconstructive oncological surgery, especially those that include microsurgery, provide various options, each with a diversity of advantages and limitations [3,4,37]. In most cases, the large soft tissue flaps (whether pedicled or not) used for palatoplasty are well-suited for the closure of the defect, but are not very suitable to support any dental prosthesis. Under these circumstances, it is almost impossible to correctly stabilize any kind of dental prosthesis without any endosseous implant, so the only option remaining is zygomatic implant placements for support [21,22,40].

In cases of reconstruction with osteo-myo-cutaneous flaps, the use of free fibula flaps (FFF) is considered the gold standard [9,11]. However, the results for maxillary bone are inferior to mandibular reconstructions, in terms of both quality of the anatomical reconstruction and dental rehabilitation. The FFF can be successfully used to reconstruct the alveolar ridge, but often it cannot be used to compensate large palatal defects. Contrarily, vascularized oste-myo-cutaneous free flaps from the hip or scapula can give a very good reconstruction of palatal defects and facial support, but they usually leave the oral cavity without any vestibule. As a result, mostly there is not enough volume or quality of bone for dental implant insertions [5,10,11]. These treatment options also represent other disadvantages, such as more invasive surgery, longer operating time, higher costs, and higher risk of failure [10,11].

Lodders et al. in 2021 evaluated outcomes from a cohort of 161 patients that suffered from cancer in the lower and mid-face, and were treated with oncologic resection and reconstruction with FFF. According to their results, approximately 5% of the group died within 6 months of the intervention, while the remaining 95% were treated as follows: 20% of the patients did not need any dental rehabilitation or were rehabilitated with removable prostheses; 28% were rehabilitated with an implant-supported prosthesis delivered after a mean period of 2 years from oncologic resection; and only 64% of these rehabilitations were successful, while the others experienced failure over a short period of function [11]. Of the overall patients, 30.5% were not rehabilitated due to complications related to the reconstructive surgery or to the oncologic pathology itself (recurrence of neoplasms in primary or secondary site), and 16% were not rehabilitated at all, due to long periods of waiting from oncologic resection to final rehabilitation [11]. Other authors reported similar results, ranging from 21% to 42.9% for receiving a functional dental rehabilitation [Iizuka et al. (2005) 21%, Smolka et al. (2008) 42.9% of the patients treated, Chiapasco et al. (2006) 28.6% and Garrett et al. (2006) 32.6%] [42,43,44,45].

The use of zygomatic implants to support the oral rehabilitation of the maxilla was initially proposed for post-oncological resections [18]. Currently, zygomatic implants are utilized as a valid treatment alternative; even in cases of very large defects in the maxillary bone, high success rates were reported [14,15,16,17,18,19,20,21,22,23,24,25,26,27]. In a study with 10 years of follow-up, Tolman reported a 96% success for zygomatic implants [53]. Similarly, Brånemark et al. reported a survival rate for zygomatic implants of 97% up to 12 years of follow-up [56]. In a more recent systematic review, Wang et al. evaluated the reliability of ZI-supported prostheses for the rehabilitation of the atrophic maxilla and reported a mean survival rate as 96.7% [57].

There are various reports in the literature regarding the rehabilitation of oncologic patients treated with tumor resection and zygomatic implants. In a 2021 review article, Hackett, El-Wazani and Butterworth analyzed the outcomes of 326 rehabilitation patients affected by cancer of the mid-face and treated with partial/hemi-/sub-total or complete maxillectomy and zygomatic implant placement [19]. According to the results, most of the patients were treated in within a 72 h to 8 months period from implant placement to oral rehabilitation. The survival rate of zygomatic implants ranged from 77% to 100% (mostly higher than 89%). In a majority of the studies, implants were placed alone as a support for obturator prostheses or in association with a soft tissue revascularized flap to give extra anchorage to the traditional fix or removable prostheses. The data demonstrated an implant failure rate around 4% when implants were placed simultaneously with the resective surgery; this percentage was 11% when they were placed in a second surgical session, and immediate loading protocols showed better results. In cases of radiotherapy, the authors were not able to reach a conclusion on timing and its role in implant failures [19]. Another systematic review on immediately loaded zygomatic implants (with a minimum follow-up of one year) reported a success rate of zygomatic implants and prostheses ranging from 96% to 100% [58].

In this study, zygomatic implant rehabilitation with fully digital surgical and prosthetic workflow was utilized for an oncologic patient. The major goals were reducing the treatment time and the invasiveness of the procedure, and to increase the quality of life of the patient. Another important aspect of the surgery was the tumor resection via endoscopic techniques. This approach, when indicated, allows the better examination of the lesion and preserves the neighboring critical anatomical structures with a safer resection margin. Furthermore, this approach avoided facial incision and the retraction of facial tissues, which leads to scars, which can affect the social and working life of the patient. To overcome several possible planning problems, and for achieving better results, a multi-skilled team is necessary, including bioengineers, dental technicians, prosthetists, and implantologists, together with ENT specialists, and oncological and microvascular surgeons [41]. The preoperative planning plays a crucial role, as the anatomical alterations caused by the oncologic resection very often eliminate all the anatomical landmarks used in free-hand zygomatic implant placement. A slight positioning or axis error of zygomatic implant placement can create a variety of problems, such as injuries in the ocular bulb, the impossibility of the placement of a preoperatively planned second zygomatic implant on the same site, or the incorrect emergence of the implant abutments, which may cause several problems in the prosthetic workflow. All these crucial factors can create limitations in the speech and function of the patients [45]. The advantages of guided surgery are evident, as it can allow perfect positioning of the zygomatic implant and accurate planning of the emergence of the implants.

In this case report, the planning protocol was designed as prosthetically driven. The extra-sinus technique was utilized for avoiding any possible negative interaction with the respiratory space, and for avoiding a perforation of the soft tissue flap [47,48]. In cases when implants are placed freehand, the loading usually takes place in the following 48–72 h post-surgery [23], due to the inevitable difficulties of working in the oral cavity of a tracheostomized patient in the lying position, and the post-operative period for sub-intensive therapy. In this case, accurate preoperative planning and coordination between the team members made it possible to achieve the construction of a fixed and stable prosthesis before surgery, that was finished and was immediately loaded directly in the operating room during general anesthesia. This allowed a satisfactory immediate rehabilitation of the patient, avoiding further subsequent interventions. As already mentioned, the prognosis of these patients is often unfortunate in the short/medium-term, and such patients would benefit from an immediate and stable rehabilitation which would improve their QoL.

In conclusion, according to the outcomes of this report, it can be stated that the use of zygomatic implants, in cases of reconstructive surgery after large resections in oncologic patients, can allow a rapid, safe, effective, and stable occlusal rehabilitation in the patients with defects extended up to class 3 of the Modified Okay classification system. These techniques can strongly improve the QoL of patients who do not have long life expectations. Guided surgery, although difficult to plan, can increase the precision and quality of the result. However, it is not possible to construct a statement based on the outcomes of a single case report, and future prospective studies with larger sample sizes and longer follow-up periods are needed. The careful planning of each case is pivotal for success and the prevention of any unexpected situations that might have a negative impact in oral rehabilitation.

## Data Availability

The authors will provide data upon request.
